# Mitochondrial Impairment by MitoBloCK-6 Inhibits Liver Cancer Cell Proliferation

**DOI:** 10.3389/fcell.2021.725474

**Published:** 2021-09-20

**Authors:** Yaschar Kabiri, Anna Fuhrmann, Anna Becker, Luisa Jedermann, Carola Eberhagen, Ann-Christine König, Tiago Barros Silva, Fernanda Borges, Stefanie M. Hauck, Bernhard Michalke, Percy Knolle, Hans Zischka

**Affiliations:** ^1^Institute of Toxicology and Environmental Hygiene, School of Medicine, Technical University of Munich, Munich, Germany; ^2^Institute of Molecular Toxicology and Pharmacology, Helmholtz Center Munich, German Research Center for Environmental Health, Neuherberg, Germany; ^3^Research Unit Protein Science and Metabolomics and Proteomics Core Facility, Helmholtz Center Munich, German Research Center for Environmental Health, Neuherberg, Germany; ^4^CIQUP, Department of Chemistry and Biochemistry, Faculty of Sciences, University of Porto, Porto, Portugal; ^5^Research Unit Analytical BioGeoChemistry, Helmholtz Center Munich, German Research Center for Environmental Health, Neuherberg, Germany; ^6^Institute of Molecular Immunology and Experimental Oncology, University Hospital Rechts der Isar, Technical University of Munich, Munich, Germany

**Keywords:** mitochondria, HCC, disulfide relay system, iron, heme

## Abstract

Augmenter of liver regeneration (ALR) is a critical multi-isoform protein with its longer isoform, located in the mitochondrial intermembrane space, being part of the mitochondrial disulfide relay system (DRS). Upregulation of *ALR* was observed in multiple forms of cancer, among them hepatocellular carcinoma (HCC). To shed light into ALR function in HCC, we used MitoBloCK-6 to pharmacologically inhibit ALR, resulting in profound mitochondrial impairment and cancer cell proliferation deficits. These effects were mostly reversed by supplementation with bioavailable hemin b, linking ALR function to mitochondrial iron homeostasis. Since many tumor cells are known for their increased iron demand and since increased iron levels in cancer are associated with poor clinical outcome, these results help to further advance the intricate relation between iron and mitochondrial homeostasis in liver cancer.

## Introduction

Hepatocellular carcinoma (HCC) is the most common form of primary liver cancers. It represents the fourth most common cause of cancer-related deaths worldwide (Kim and Viatour, 2020). Although Hepatitis B and C are regarded as the leading cause of HCC, the sharp rise in non-alcoholic fatty liver disease (NAFLD) as well as alcoholic and non-alcoholic steatohepatitis (ASH/NASH) are becoming strong contributing factors for the remarkable increase in HCC incidence in Western Countries (Anstee et al., 2019). HCC is usually diagnosed in advanced tumor stages because of its long asymptomatic window. This, combined with the often underlying liver cirrhosis, strongly limits treatment options and mostly rules out curative approaches (Llovet et al., 2008). HCC is notoriously resistant to chemical and radiation treatments. Emerging chemical treatments are currently limited to tyrosine kinase inhibitors with often questionable benefit and strong co-toxicity (Anstee et al., 2019).

Over the last decades, research has shown that mitochondria are very promising targets in cancer therapy due to the reprogrammed metabolism in many tumors (Ward and Thompson, 2012). This was recently demonstrated in HCC models, in which treatment by the antibiotic tigecycline that impairs mitochondrial protein synthesis, was able to overcome the massive tumor relapse upon sorafenib withdrawal (Messner et al., 2020). Development of such second-line treatments targeting cancer mitochondria thus might be highly valuable since tyrosine kinase inhibitor treatment is of a short-lived nature due to dose-limiting toxicity and quickly developing resistances in HCC (van Malenstein et al., 2013).

Along this line, one mitochondrial metabolic pathway that is upregulated in a number of different cancers is the MIA40/ALR import and folding machinery, also known as the disulfide relay system (DRS) (Thomas and Ashcroft, 2019). This unique mitochondrial system oxidatively folds a specific group of cysteine-motif containing substrates and subsequently traps them in the mitochondrial intermembrane space. Electrons from the oxidized substrate proteins reduce two cysteine residues in MIA40, which transfers them to the augmenter of liver regeneration (ALR) for its regeneration. ALR passes these electrons to cytochrome C to feed them into the respiratory chain (Fischer and Riemer, 2013). Numerous studies point out that ALR is upregulated in HCC, suggesting a pivotal role of this relay system in this tumor type [reviewed in Nguyen et al. (2017)]. ALR is an ubiquitously expressed protein with two main isoforms, a shorter 16 kD protein and a longer 23 kD protein which contains a mitochondrial targeting sequence. Only the latter is part of the DRS in the mitochondrial intermembrane space (Ibrahim and Weiss, 2019). Mutations in the corresponding gene are associated with alterations in mitochondrial ultrastructure and respiratory chain deficiencies (Di Fonzo et al., 2009). Since the DRS is essential for correct function of the mitochondrial respiratory chain, we concluded that this pathway might pose a promising anti-cancer target and its investigation in HCC might prove insightful.

Full ALR knockouts are lethal because of its essential role, leaving knockdowns as an alternative (Rissler et al., 2005). However, since current knockdowns affect all ALR isoforms, it is unknown if the observed effects can be attributed to one specific isoform (Ibrahim and Weiss, 2019). On the other hand, Dabir et al. (2013) described a small molecule, termed MitoBloCK-6 (MB-6) that specifically inhibited Erv1p function in yeast mitochondria. Erv1p is essential for respiration, resides in the mitochondrial intermembrane space, and ALR is its mammalian ortholog (Lange et al., 2001; Hofhaus et al., 2003). Thus, while also in yeast there are enzymatic isoforms, as a second sulfhydryl oxidase, termed Erv2p, has been identified in the endoplasmic reticulum (Gerber et al., 2001), functional mitochondrial impairments upon MB-6 exposure are plausibly related to Erv1p/ALR. Importantly, MB-6 induced cell death in human embryonic stem cells but not in differentiated cells. Recently, Singh et al. (2020) demonstrated that MB-6 reduced growth and viability of acute myeloid lymphoma stem cells while impairing mitochondrial structure and function.

We thus utilized MB-6 to block ALR in HCC cells. In this study, we demonstrate that MB-6 strongly inhibits HCC cell proliferation and leads to various mitochondrial impairments. These effects are mostly rescued by supplying the cells with non-toxic concentrations of bioavailable hemin, adding evidence toward a – much-debated – role of ALR in cellular iron metabolism and cell fate.

## Materials and Methods

### Cell Culture and Treatments

The rat hepatocellular carcinoma cell line McA-RH7777 (McA) was obtained from ATCC and was cultured in Dulbecco’s Modified Eagle Medium (DMEM) with 1.9 mM Glutamax and 4.5 g/L Glucose, supplemented with 10% FCS Superior (Bio&SELL, Germany) and 1% Penicillin/Streptomycin (Gibco, United Kingdom). Cells were maintained at 37°C in an atmosphere with 95% humidity and 5% CO_2_. Cell number was determined with a Neubauer counting chamber for passaging or with an automated cell counter (LUNA-II, Logos Biosystems) for seeding experiments.

For treatments, 1 ^∗^ 10^6^ McA cells were seeded into 10 cm plates and incubated for 72 h. Cells were treated with 30 μM MB-6 for 72 h unless indicated otherwise. Cells for mitochondrial network and ultrastructure analyses were treated with both 20 and 40 μM MB-6 for 72 h to better highlight structural differences. For rescue experiments, cells were co-treated with 1.17 μM methemalbumin. Methemalbumin was prepared as described previously (Michener et al., 2012), albumin-bound protoporphyrin-IX and chlorophyll were prepared analogously.

### Proliferation Measurements

To determine cellular proliferation, 1 ^∗^ 10^5^ McA cells were seeded in 6-well plates and incubated in the presence or absence of MB-6 for 72 h. Cells were then trypsinized and cell number as well as cell size were determined via automated cell counting.

### Cytotoxicity and Combined Growth and Viability Measurements

To determine acute cytotoxicity, 2 ^∗^ 10^4^ cells were seeded in 96-well plates in the presence or absence of treatment substances and incubated for 24 h. To determine combined growth and viability, 5 ^∗^ 10^3^ cells were seeded in 96-well plates in the presence or absence of treatment substances and incubated for 72 h. Afterward, cell viability was measured via CellTiter-Glo^®^ (Promega, Germany).

### Cell Cycle Analysis

Cell cycle analysis was done as described (Pozarowski and Darzynkiewicz, 2004). Briefly, cells were treated for 48 h and then trypsinized. 1 ^∗^ 10^6^ cells were suspended in PBS and fixated by dropwise addition to ice-cold 70% ethanol. Thereafter, cells were washed with PBS and stained with propidium iodide solution, also containing RNase A, for 30 min. Cells were then analyzed with a FACS Canto II (BD Dickinson, United States).

### Semi-Thin Sections and Electron Microscopy

Preparation of semi-thin sections and ultra-thin sections of cells was performed as described previously (Zischka et al., 2008; Schulz et al., 2013). Semi-thin sections were imaged with a Nikon Eclipse Ti-S with NIS-Elements (Nikon, Japan). Electron micrographs were imaged with 1200EX electron microscope (JEOL, Japan). Images were acquired with a KeenView II digital camera (Olympus, Germany) and processed via iTEM (analySIS FIVE, Olympus, Germany). For mitochondrial structure analysis, at least 100 mitochondria per condition were analyzed and assigned to groups based on their ultrastructure. Analysis was done on biological triplicates.

### Mitochondrial Isolation

Mitochondria from cultured cells were isolated as described previously (Schmitt et al., 2013; Kabiri et al., 2021b). Briefly, 5 ^∗^ 10^6^ cells/mL were pumped through the pump-controlled rupture system three times at 6 μm clearance and 1,000 μL/min flow rate. The resulting homogenate was centrifuged at 1,000 × *g* for 10 min at 4°C to remove nuclei and debris. The supernatant was centrifuged at 10,000 × *g* for 10 min at 4°C to yield crude mitochondria. For proteomic analyses, crude mitochondria were further purified via Nycodenz gradient centrifugation (24%/18%) in a swing-out ultracentrifuge at 95,000 × *g* for 15 min at 4°C.

### Enzyme Activities

Complex I and II activities were measured as described (Spinazzi et al., 2012), modified to be measured in 96-well plates. For complex I measurement, mitochondria were freeze-thawed three times with liquid nitrogen to disrupt the mitochondrial membranes. 10 μg of crude mitochondria per well were incubated for 10 min with or without rotenone with a premix containing all chemicals except NADH. NADH was added and absorbance was recorded at 600 nm for 5 min at 37°C. The remaining rotenone-insensitive activity was subtracted from total activity to yield rotenone-sensitive complex I activity. For complex II measurement, 20 μg of crude mitochondria per well were incubated for 5 min with or without thenoyltrifluoroacetone with a premix containing all chemicals except decylubiquinone. Decylubiquinone was added and absorbance was recorded at 600 nm for 10 min at 37°C.

Aconitase activity was assessed as described (Schulz et al., 2006). Briefly, crude mitochondria were freeze-thawed three times in liquid nitrogen. Mitochondria were then assayed in the presence of reaction buffer and NADP^+^ formation was recorded at 340 nm for 60 min at 37°C.

### Cellular and Mitochondrial Redox Status

Cellular and mitochondrial glutathione status was determined as described (Rahman et al., 2006) with minor modifications. Crude cells or mitochondria were treated with equal volumes of 10% metaphosphoric acid and sonicated to precipitate proteins, followed by neutralization of residual acid with triethanolamine. Total GSH content of the samples was measured without further preparation. GSSG content was measured by treating samples with 2-vinylpyridine to mask GSH. Samples were measured in reaction buffer after addition of 5,5′-Dithiobis-2-nitrobenzoic acid at 412 nm for 10 min. NAD^+^/NADH levels and NADPH/NADP^+^ levels were measured with the NAD/NADH-Glo^TM^ and NADPH/NADP-Glo^TM^ assay respectively, according to the manufacturer’s instructions (Promega, Germany).

### Mitochondrial Heme Content

Mitochondrial heme concentration was determined as described previously (Michener et al., 2012). Briefly, crude mitochondria were pelleted, resuspended in 20 mM oxalic acid and stored in the refrigerator for at least 24 h. Subsequently, equal volumes of 2 M oxalic acid were added and samples were split. One aliquot was kept untreated for determination of background fluorescence and one aliquot was heated to 98°C for 30 min. Fluorescence was measured at λ_*ex*_ = 400 nm and λ_*em*_ = 620 nm. Heme concentrations were calculated using a hemin standard curve.

### Proteomic Analysis

#### Sample Preparation

10 μg of purified mitochondria were subjected to a proteolysis applying a modified filter aided sample preparation (FASP) procedure (Wisniewski et al., 2009; Grosche et al., 2016). After protein reduction and alkylation using DTT and iodoacetamide, samples were denatured in UA buffer (8 M urea in 0.1 M Tris/HCl pH 8.5) and centrifuged on a 30 kDa cut-off filter device (PALL or Sartorius) and washed thrice with UA buffer and twice with 50 mM ammonium bicarbonate (ABC). Proteins were proteolysed for 2 h at room temperature using 0.5 μg Lys-C (Wako) and subsequently for 16 h at 37°C using 1 μg trypsin (Promega). Peptides were collected by centrifugation and acidified with 0.5% trifluoroacetic acid (TFA).

#### Mass Spectrometric Measurements

LC-MSMS analysis was performed on a Q-Exactive HF mass spectrometer (Thermo Scientific) online coupled to a nano-RSLC (Ultimate 3000 RSLC; Dionex). Tryptic peptides were accumulated on a nano trap column (300 μm inner diameter × 5 mm, packed with Acclaim PepMap100 C18, 5 μm, 100 Å; LC Packings) and then separated using reversed phase chromatography (nanoEase MZ HSS T3 Column, 100Å, 1.8 μm, 75 μm × 250 mm; Waters) in a 80 min non-linear gradient from 3 to 40% acetonitrile (ACN) in 0.1% formic acid (FA) at a flow rate of 250 nl/min. Eluted peptides were analyzed by the Q-Exactive HF mass spectrometer equipped with a nano-flex ionization source. Full scan MS spectra (from m/z 300 to 1,500) and MSMS fragment spectra were acquired in the Orbitrap with a resolution of 60,000 or 15,000 respectively, with maximum injection times of 50 ms each. The up to ten most intense ions were selected for HCD fragmentation depending on signal intensity (TOP10 method). Target peptides already selected for MS/MS were dynamically excluded for 30 s.

#### Progenesis QI for Label-Free Quantification

Spectra were analyzed using Progenesis QI software for proteomics (Version 3.0, Non-linear Dynamics, Waters, Newcastle upon Tyne, United Kingdom) for label-free quantification as previously described (Grosche et al., 2016). All features were exported as Mascot generic file (mgf) and used for peptide identification with Mascot (version 2.4) in the Ensembl Rat database (Release 2014.02, 28611 sequences). Search parameters used were: 10 ppm peptide mass tolerance and 20 mmu fragment mass tolerance, one missed cleavage allowed, carbamidomethylation was set as fixed modification, methionine oxidation and asparagine or glutamine deamidation were allowed as variable modifications. A Mascot-integrated decoy database search calculated an average false discovery of <1%.

#### Mitominer Analysis

Mitochondrial assignment of proteins was assessed using Mitominer 4.0. Analysis was performed based on mouse gene symbols.

### High-Resolution Respirometry

Mitochondrial respiration was measured in an Oxygraph-2k with DatLab 7.4 (Oroboros Instruments, Innsbruck, Austria). 2 ^∗^ 10^6^ cells per chamber were added to 2.0 mL Mir05 buffer (0.5 mM EGTA, 3 mM MgCl_2_, 60 mM lactobionic acid, 20 mM taurine, 10 mM KH_2_PO_4_, 20 mM HEPES, 110 mM sucrose, 1 g/l albumin, pH 7.1) to measure routine respiration. 5 μg/mL digitonin was added to fully permeabilize the cells. NADH-linked respiration was measured after addition of 5 mM pyruvate, 2 mM malate, and 2.5 mM ADP. Afterward, 0.5 μM rotenone and 10 mM succinate were added to determine succinate-linked respiration. Residual oxygen consumption was determined by final addition of 2.5 μM antimycin A.

Complex III-linked respiration was measured by adding cells and digitonin as described above, followed by addition of 0.5 μM rotenone, 5 mM malonate and 2.5 mM ADP. Respiration was initiated by addition of 0.5 mM duroquinol, an artificial complex III substrate. Thereafter, residual oxygen consumption was measured by addition of 2.5 μM antimycin A.

### Metal Analysis

Cellular and mitochondrial iron content were analyzed by ICP-OES (Spectro ARCOS, SPECTRO Analytical Instruments, Kleve, Germany) as described previously (Zischka et al., 2011; Einer et al., 2019). Briefly, either 2.5 ^∗^ 10^6^ cells or 300 μg mitochondria were washed with PBS, resuspended in 1.5 mL ddH_2_O and mixed with equal volumes of suprapure HNO_3_ (Supelco Inc., United States). Samples were stored for at least 24 h before being subjected to optical emission spectrometry.

### Fluorescence Microscopy

Cells were cultivated for 72 h on 8-well glass bottom microslides (ibidi, Germany) coated with Poly-D-lysine. Afterward, cells were stained with 75 nM MitoTracker^TM^ Green and MitoTracker Deep Red (Thermo Scientific) for 30 min and with 1 μg/mL Hoechst^®^ 33342 (Thermo Scientific) for 5 min, followed by two washing steps with PBS. After staining, cells were kept in cell culture media and were imaged on an ECLIPSE T*i*-S fluorescence microscope (Nikon, Japan) at 600× magnification.

### Statistics

Data are represented as either individual or mean values with standard deviation (SD). All experiments were at least performed in triplicates. All data were tested for Gaussian distribution and outlier analysis was performed by the ROUT method at (*Q* = 1%). Statistical significance was analyzed using either 1-way ANOVA with or without Dunnet’s multiple comparison test or by using unpaired two-tailed *t*-test. All data analyses were performed using GraphPad Prism 8 (GraphPad Software Inc., United States). Statistical differences are expressed as ^∗^*p* < 0.05, ^∗∗^*p* < 0.01, ^∗∗∗^*p* < 0.001, and ^****^p < 0.0001.

## Results

### MitoBloCK-6 Provokes Pronounced Proliferation Deficits in Hepatocellular Carcinoma Cells

Upregulation of the mitochondrial disulfide relay (DRS) in many different cancers makes it a promising potential target in cancer therapy (Nguyen et al., 2017). However, since knockouts of the DRS are lethal, this system needs to be targeted either by knockdown or chemical inhibition (Rissler et al., 2005). ALR, which regenerates the thiol groups in mitochondrial MIA40, is present in various isoforms with entirely different functions and cellular localizations, complicating a knockdown. Until recently, studies have not discriminated between these isoforms when knocking down ALR, thereby further impeding result interpretation. We therefore decided to chemically inhibit ALR in liver cancer using MitoBloCK-6 (MB-6). As MB-6 interferes with yeast as well as human cancer mitochondria (Dabir et al., 2013; Singh et al., 2020), this approach offers a reasonable extent of specific subcellular compound activity and therefore a most plausible action on the mitochondrial ALR isoform.

**FIGURE 1:**
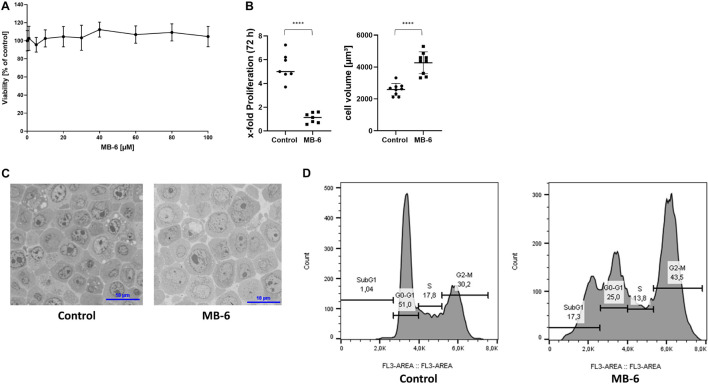
MB-6 negatively affects McA-RH7777 cells in morphology and proliferation. **(A)** 24 h treatment of cells with MB-6 showed no signs of acute cytotoxicity in concentrations up to 100 μM. Higher concentrations would result in crystallization of MB-6. **(B)** 72 h treatment of cells with 30 μM MB-6 decreased cellular proliferation close to a complete proliferation arrest along with a near doubling in cell volume. **(C)** Micrographs from semi-thin sections of cells treated for 72 h with 30 μM MB-6 demonstrate an increase in cellular volume as well as multiple and fragmented nuclei. Scale bars equal 10 μm. **(D)** Cell cycle analysis of fixed cells reveals a strong increase in the SubG1 and G2-M cell population with a concomitant decrease in G1 population. ****p < 0.0001.

To investigate acute cytotoxicity, we incubated the rat HCC cell line McA-RH7777 (McA) with different concentrations of MB-6 over 24 h. This HCC cell line was chosen because previous proteomic studies in our group have shown upregulation of the DRS compared to healthy rat liver mitochondria (data not shown). MB-6 did not show signs of direct cytotoxicity over a time span of 24 h in concentrations of up to 100 μM (Figure 1A). Higher concentrations caused MB-6 to precipitate and were thus excluded. Upon 72 h of exposure however, MB-6 strongly attenuated cell growth, accompanied by an almost twofold increase in cell volume. Cell growth decreased from fivefold proliferation over 72 h to less than twofold proliferation in the same time span (Figure 1B). This strong anti-proliferative property of MB-6 is in accordance with a very recent study in AML cells (Singh et al., 2020). The increase in cell size was accompanied by nuclear enlargement (Figure 1C). Cell cycle analysis of fixed cells revealed that MB-6 treatment resulted in a cell cycle block at G2-M phase and a marked increase of SubG1 populations indicating cell death to occur at 72 h of incubation (Figure 1D).

### MitoBloCK-6 Alters Mitochondrial Ultrastructure and Strongly Elevates Heme Synthesis Proteins in Hepatocellular Carcinoma Mitochondria

**FIGURE 2:**
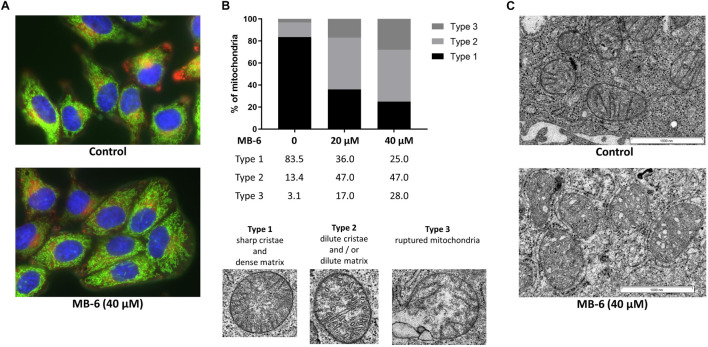
MB-6 impairs the mitochondrial ultrastructure. **(A)** Fluorescence imaging of cells stained with Hoechst^®^ 33342, MitoTracker^TM^ Green FM and LysoTracker^TM^ Deep Red shows intactness of the mitochondrial tubular network as well as no increased lyosomal activity. **(B)** Quantitative analysis of mitochondria demonstrates that MB-6 causes dose-dependent decline in type 1 mitochondria (sharp, elongated cristae) along with an increase in type 2 (rounded, ballooned cristae) and type 3 (ruptured membranes) mitochondria. Type 1 resembles mitochondria with an electron-dense matrix and crisp, tubular cristae; type 2 mitochondria show enlarged, bloated cristae and/or a decreased matrix density; type 3 mitochondria possess ruptured outer membranes and are thus considered non-functional. **(C)** Electron micrographs of MB-6 treated cells reveal alterations in mitochondrial ultrastructure. Treatment results in a concentration-dependent increase in mitochondria with diluted, ballooned cristae and/or disrupted cristae structure along with a decrease in mitochondria with sharply defined, dense cristae. Scale bars equal 1,000 nm.

Next we investigated the treated cells’ mitochondrial network structure. To this end, cells were stained with MitoTracker^TM^ Green FM and LysoTracker^TM^ Deep Red upon MB-6 treatment. Neither a difference in mitochondrial network formation nor in lysosomal recruitment was observed up to 40 μM MB-6 (Figure 2A). We thus expanded mitochondrial structure analysis to ultrastructural level via electron microscopy. Indeed, electron micrographs revealed that a majority of mitochondria presented an altered ultrastructure in an MB-6 dose-dependent manner. To quantify these alterations, mitochondria were classified as one of three distinct types: type 1 with sharp cristae and an electron dense matrix; type 2 with dilute cristae and/or dilute matrix structure, and type 3 with a ruptured outer membrane (Figure 2B). While in untreated cells, more than 80% of all classified mitochondria were of the highly structured type 1, only 28% of all mitochondria were still classified as type 1 in the 40 μM treatment, whereas 47% were type 2 and 28% were type 3 (damaged) mitochondria, respectively (Figures 2B,C). Most importantly, at the postulated site of MB-6 action, i.e., the mitochondrial intermembrane space, we could clearly observe structural alterations in the form of pronounced cristae ballooning (Figure 2C) in line with very recent observations in MB-6-treated AML cells (Singh et al., 2020).

Next, we investigated the mitochondrial proteome upon cellular MB-6 treatment. To this end, we treated cells for 72 h and isolated mitochondria via the PCC method with a subsequent density gradient purification developed in our group (Schmitt et al., 2013; Kabiri et al., 2021a, b). This analysis revealed a total of 144 significantly elevated and 18 depleted proteins in mitochondria from MB-6 treated versus untreated cells (Tables 1, 2). The majority of proteins with increased abundance are involved in mitochondrial translation, biogenesis and hormesis, as well as in the assembly and function of the respiratory chain. Moreover, 36 out of 42 known DRS substrates could be quantitatively detected (Supplementary Figure 1). Out of these, five were significantly elevated (>1.5-fold), 30 were not regulated differentially and only one, CHCHD10, was depleted by more than 0.5-fold (Supplementary Figure 1). Thus, MB-6 treatment did not result in broad mitochondrial DRS substrate depletion in HCC mitochondria. Rather, specific mitochondrial pathways were targeted as, among the most upregulated proteins, four were major elements of the mitochondrial heme biosynthesis (Table 1). Of these, 5-aminolevulinate synthase (ALAS1), the rate-limiting enzyme in heme biosynthesis (Riddle et al., 1989), was increased ∼12-fold (Table 1). The most-elevated protein was SLC25A39, which is a key component of early heme biosynthesis in yeast, zebrafish, and mammals.

**TABLE 1:** Grouped list of mitochondrial proteins upregulated by MB-6 treatment.

	Fold upregulation
**Heme/porphyrin biosynthesis**	
Slc25a39	31,18
Alas1	12,36
Ppox	1,51
Tspo	1,73
**Mitochondrial ribosomes**	
Mrps33	1,80
Mrpl27	1,77
Mrps21	1,73
Mrpl14	1,69
Mrps30	1,69
Mrps34	1,68
Mrpl51	1,65
Mrps17	1,65
Mrpl15	1,56
Mrps5	1,55
Mrpl2	1,54
Mrpl20	1,66
Mrpl52	1,62
**Mitochondrial transcription/translation**	
Qtrtd1	17,22
Mrm1	3,97
Mtg2	3,63
Bpnt1	2,67
Tk2	2,62
Top1mt	2,45
Gtpbp3	2,42
Mto1	2,31
Vars2	2,28
Tfb1m	2,19
Mrs2	2,17
Polg	2,16
Thnsl1	2,07
Mterf	1,98
Ddx28	1,97
Mtpap	1,86
Pdf	1,82
Polrmt	1,81
Gfm2	1,69
Dhx30	1,67
Chdh	1,66
Fastkd1	1,62
Supv3l1	1,60
Tfb2m	1,53
Fars2	1,50
**Mitochondrial hormesis and biogenesis**	
Lyrm1	2,83
Tmem65	2,65
Mff	2,64
Abcb10	2,61
Pgs1	2,29
Tomm40l	2,24
Agpat5	2,02
Fastkd3	1,95
Timm17b	1,95
Slc25a46	1,88
Gpt2	1,82
Mthfd2l	1,77
Lonp1	1,75
Rhot2	1,68
Slc25a4	1,67
Armc10	1,66
Mpv17	1,61
Bcs1l	1,59
Cyb5r1	1,59
Yme1l1	1,58
LOC100362432	1,56
Slc30a9	1,54
Slc25a11	1,54
Mthfd1l	1,53
Tmem11	1,53
Slc25a25	1,52
Ak3	1,50
**OXPHOS assembly and maturation**	
Sfxn3	3,01
Timmdc1	2,39
Cox17	2,30
LOC100910689	2,11
Tmem126b	2,06
Foxred1	1,83
Coq4	1,78
Iba57	1,74
Cox16	1,73
Cyb5b	1,68
Tbrg4	1,65
LOC100911779	1,64
Coq7	1,62
Fmc1	1,61
**(Pro)apoptosis**	
Bak1	2,38
Bnip3l	2,23
Fam162a	2,06
Mtch1	1,90
Bcl2l13	1,84
Bcl2l1	1,65
**Catabolism**	
Lipt2	8,09
Hk1	5,39
Sqrdl	5,03
Mpc1	2,90
Slc25a24	2,87
Slc25a45	2,61
Pdk3	2,57
Aadat	2,29
Hdhd3	2,22
Kmo	2,18
Gls	2,18
Abhd10	2,07
Sirt5	1,96
D2hgdh	1,95
Sdr39u1	1,86
Echdc1	1,84
Aldh5a1	1,81
Acot9	1,77
Pck2	1,76
Crat	1,75
Bckdk	1,74
Me2	1,66
LOC100910173	1,66
Flad1	1,59
Nln	1,56
Gls	1,56
Abhd11	1,55
Gpd2	1,54
Acot2	1,53
Cpt1a	1,50
Agk	1,50
**Miscellaneous and unknown**	
Mcart1	3,86
Nif3l1	3,75
Nlrx1	3,42
Pnpla8	2,98
Vwa8	2,97
Slc25a45	2,61
Dhrs1	2,00
Mettl15	1,96
Hsdl1	1,93
Rdh14	1,85
Spr	1,82
Adck1	1,80
Tmem186	1,80
Ccdc127	1,77
Micu1	1,75
Nt5dc3	1,74
Lyrm2	1,72
Slc25a44	1,68
Micu2	1,57
Afg3l2	1,55
Ociad1	1,55
Afg3l2	1,55

**TABLE 2:** Proteins downregulated by MB-6 treatment.

Cisd2	0,46
Snd1	0,44
Lrrc59	0,44
Acsl5	0,43
Slc27a2	0,40
Chchd10	0,39
Hsp90b1	0,37
Rcn2	0,33
Pdia3	0,29
Cyb5a	0,29
Nucb2	0,27
P4hb	0,26
Emc2	0,25
Prdx4	0,23
Myh9	0,22
Hyou1	0,20
Glul	0,18
Tm9sf4	0,10

### MitoBloCK-6 Disrupts Cellular Iron Homeostasis and Redox Status in Hepatocellular Carcinoma

**FIGURE 3:**
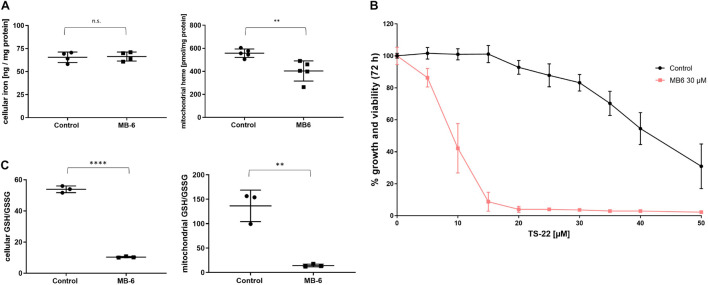
MB-6 treatment lowers mitochondrial heme levels as well as cellular and mitochondrial GSH/GSSG ratios and results in increased sensitivity to iron chelators. **(A)** MB-6 treatment does not alter total cellular iron content (left) but significantly decreases total mitochondrial heme content by almost 30% (right). **(B)** Co-treatment with the iron chelator TS-22 reveals increased sensitivity of MB-6 treated cells toward iron depletion. **(C)** Both cellular (left) and mitochondrial **(right)** GSH/GSSG ratios are significantly decreased in MB-6 treated cells. ^∗∗^*p* < 0.01, ^****^p < 0.0001.

As the elevated presence of heme biosynthesis proteins may be linked to a possible impairment in mitochondrial iron status, mitochondria from MB-6 treated cells were subjected to metal analysis. This revealed that the cellular total iron as well as the mitochondrial iron contents were not influenced by MB-6 treatment (Figure 3A and Supplementary Figure 4). To discriminate between iron species, total heme in isolated mitochondria was quantified. In contrast to the unchanged iron levels, mitochondrial heme levels from MB-6 treated samples were significantly lower than in control mitochondria (Figure 3A). Thus, it was not the mitochondrial iron *per se* but rather its biochemically active form that was negatively influenced by MB-6. We therefore decided to further interfere with mitochondrial heme synthesis and decreased the cells’ iron supply by co-treatment with organic iron chelators. Thereto, either a synthetic derivative of deferiprone, termed TS-22, containing an α-hydroxyketone motif or the standard iron chelator deferoxamine (DFO) were employed. Indeed, co-treatment of these iron chelators with MB-6 resulted in massive cellular growth and viability deficits (Figure 3B and Supplementary Figure 2B). Thereat, DFO was slightly less effective than TS-22, as DFO treatment alone (i.e., without MB-6) already proved to be more toxic than TS-22 (Supplementary Figure 2B). As biochemically active iron (i.e., heme bound) was reduced in MB-6 mitochondria but total iron was not, we further reasoned that free iron species maybe elevated and could therefore provoke elevated oxidative stress via Fenton-based chemistry (Winterbourn, 1995). We thus investigated the cellular and mitochondrial redox status upon MB-6 treatment. After 72 h of treatment, the cellular GSH/GSSG ratio decreased from 54:1 to 10:1. This effect was even more pronounced in mitochondria, in which the GSH/GSSG ratio dropped from 136:1 to 14:1 (Figure 3C). The ratios of other important cellular redox pairs, NAD+/NADH and NADPH/NADP+ on the other hand, remained unaffected (Supplementary Figure 2A).

### MitoBloCK-6 Affects Selected Iron-Containing Proteins in Hepatocellular Carcinoma Mitochondria

**FIGURE 4:**
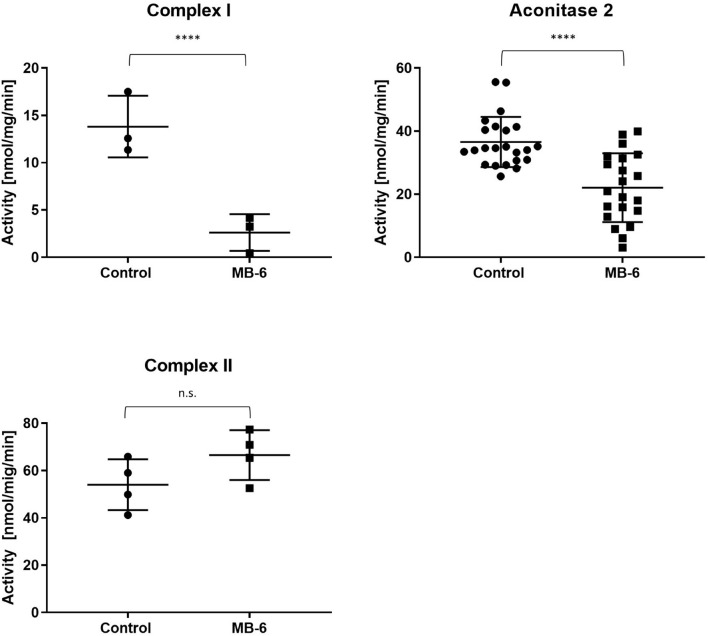
Selected Fe-S cluster protein activities are negatively affected by MB-6 treatment. Upon MB-6 treatment, complex I activity as well as aconitase II activity drop significantly, whereas complex II activity remains unchanged. ^****^p < 0.0001.

The disturbed iron homeostasis and increased sensitivity of MB-6 treated cells to iron chelators led us to investigate the activities of several iron-containing mitochondrial proteins. Out of three such proteins, complex I was the most severely affected with its rotenone-sensitive activity decreasing from 14 to 2.6 nmol/mg/min (Figure 4). Aconitase 2 was less impaired with its activity decreasing from 37 to 22 nmol/mg/min (Figure 4). Lastly, complex II activity was unaffected by MB-6 treatment (Figure 4).

### Bioavailable Hemin Mostly Rescues MitoBloCK-6 Affected Mitochondrial Respiration and Ultrastructure in Hepatocellular Carcinoma Mitochondria

**FIGURE 5:**
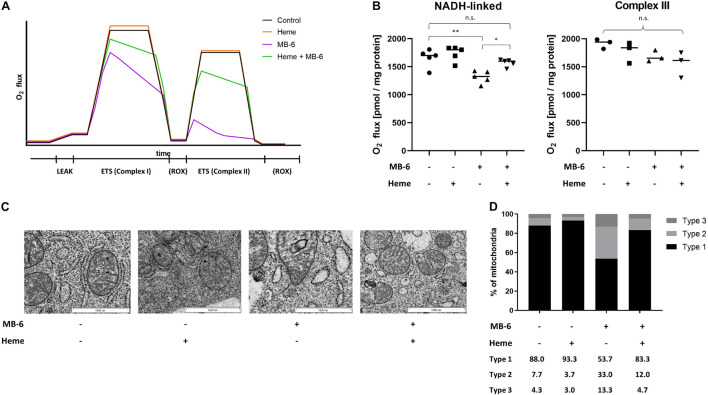
MB-6 impaired mitochondrial function and structure are both strongly improved by methemalbumin supplementation. **(A)** Representative presentation of high-resolution respirometry with both NADH-linked and FAD-linked substrates. Respiration of control cells (black) and heme-treated cells (orange) are indistinguishable. MB-6 treatment (violet) results in lower initial maximum respiration and causes a steady decline in O_2_ flux over time which carries over to succinate-linked respiration. Double treatment (green) significantly restores initial respiration and alleviates the continuous decline in O_2_ flux. LEAK and ROX respirations are comparable in all four conditions. **(B)** NADH-linked peak respiration is strongly impaired by MB-6 treatment. Methemalbumin supplementation rescues the impairment almost entirely. Meanwhile, complex III-driven respiration fueled by an exogenous ubiquinol derivative remains unchanged by either treatment. ^∗^*p* < 0.05, ^∗∗^*p* < 0.01. **(C)** Electron micrographs reveal that mitochondrial ultrastructure is almost restored to control levels upon methemalbumin supplementation. **(D)** Quantitative analysis of mitochondria demonstrates a significant decline in type 2 and type 3 mitochondria close to control conditions.

The MB-6 provoked impairment of complex I activity was further validated by high-resolution respirometry (HRR) of treated cells. MB-6 treatment led to a statistically significant decline in peak NADH-linked respiration from 1723 ± 53 pmol/mg/s to 1317 ± 109 pmol/mg/s. This reduced oxygen flux continued to decrease over time, even upon addition of complex II substrates (Figure 5A), indicating progressive mitochondrial failure (Figure 5A). However, if respiration was fueled directly via complex III with the artificial substrate duroquinol, oxygen flux from MB-6 treatment remained unchanged (Figure 5B). This demonstrates a pronounced and progressive respiratory deficit in mitochondria from MB-6 treated cells and, together with the enzymatic deficits in complex I, suggests the initial parts of the electron transport chain to be severely impaired.

As MB-6 impaired the mitochondrial heme homeostasis, we supplemented MB-6 treated cells with iron in the form of hemin. Since free hemin is not cell-permeable and thus not bioavailable, methemalbumin was used as hemin carrier (Muchova et al., 2015). This supplementation caused a massive and significant increase in mitochondrial iron (Supplementary Figure 5A) demonstrating its correct delivery. Furthermore, it was delivered biochemically functional as a profound rescue effect on NADH-linked respiration was observed (Figures 5A,B). Initial oxygen flux upon ADP addition increased to 1569 ± 66 pmol/mg/s, which was statistically insignificant from untreated cells as well as cells treated with methemalbumin alone. Furthermore, the time-dependent steady decline in oxygen flux resulting from MB-6 treatment clearly stabilized if cells were supplemented with methemalbumin. Additionally, this double treatment resulted in a pronounced improvement of mitochondrial ultrastructure. The proportions of mitochondria with well-defined, linear cristae (type 1) as well as mitochondria with altered cristae and/or matrix structure were almost restored to normal. The increase in ruptured mitochondria was completely alleviated by double treatment (Figures 5C,D). Finally, MB-6 induced cell death was significantly reduced by methemalbumin supplementation, as we observed a strong decrease in SubG1 cell populations (Supplementary Figure 3).

### Alleviation of MitoBloCK-6 Inhibition in Hepatocellular Carcinoma Mitochondria Is Specific to Methemalbumin Supplementation

**FIGURE 6:**
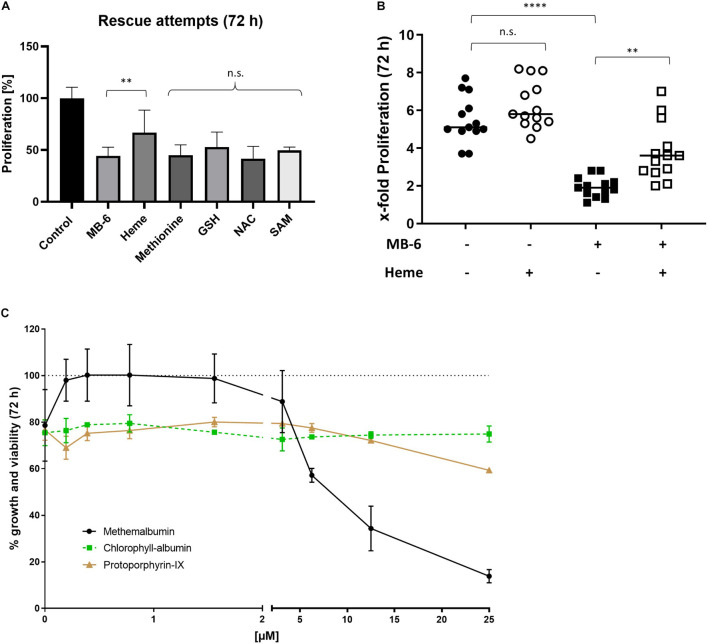
Proliferation inhibition by MB-6 can be partially rescued by methemalbumin supplementation but not by closely related heme analogs. **(A)** Proliferation is rescued by providing cells with heme in the form of methemalbumin but not by providing various means of restoring the impaired GSH/GSSG status. **(B)** Methemalbumin addition partially rescues cellular proliferation of MB-6 treated cells but does not affect cellular proliferation on its own. **(C)** The described rescue effect is specific to methemalbumin. Neither protoporphyrin-IX-albumin (lacking the iron), nor chlorophyll-albumin (containing redox-active Mg instead of iron), improve the impairment in growth in viability caused by MB-6. Note that methemalbumin becomes toxic at higher concentrations. **p < 0.01, ****p<0.0001.

MB-6 caused a profound shift in GSH/GSSG ratios toward GSSG (Figure 3C). Previous reports demonstrated that GSH plays a crucial role in correct cysteine oxidation facilitated by the DRS and that GSH levels need to be carefully balanced (Mesecke et al., 2005; Bien et al., 2010). Thus, we attempted to rescue MB-6 treated cells with either GSH directly or various GSH precursors, namely methionine, *N*-acetylcysteine (NAC) and *S*-adenosylmethionine (SAM). Neither co-treatment had a significant effect on proliferation (Figure 6A). In contrast, only supplementation with methemalbumin rescued the profound proliferation inhibition caused by MB-6 treatment (Figure 6B). This rescue effect was specific to heme-bound iron, as neither protoporphyrin-IX, a heme precursor devoid of iron, nor chlorophyll A, a heme analog with magnesium as a central ion, up to tested concentrations of 25 μM had any rescue effect on growth and viability. Methemalbumin on the other hand effectively rescued MB-6 induced growth and viability inhibition at concentrations between 0.3 and 2 μM. At higher concentrations it became cell toxic itself (Figure 6C). Finally, we investigated whether this co-treatment was able to improve the impaired mitochondrial GSH/GSSG ratios. While a positive tendency was noted here, this treatment nevertheless failed to restore this ratio significantly (Supplementary Figure 4B).

## Discussion

The mitochondrial DRS is an essential part of protein maturation in eukaryotic cells, highlighted by the finding that mutations in the ALR gene are associated with mitochondrial myopathies and impairment of the respiratory chain (Di Fonzo et al., 2009; Nambot et al., 2017). Its differential regulation in various malignancies is well-documented, warranting investigation about its implications in cancer. Particularly, a concisely reported upregulation of the DRS in HCC (Yu et al., 2014), focused our interest in the present study.

However, previous studies on ALR rarely discriminated between its isoforms, the mainly cytosolic short form, and the predominantly mitochondrial long form (Nguyen et al., 2017). These two isoforms perform vastly different functions, making any results including non-isoform specific knockdown and overexpression hard to interpret. Furthermore, ALR inhibition seems to have tissue/cell-type dependent effects. For example, in a recent study, Singh et al. (2020) reported, that inhibiting ALR in AML cells caused an increase in mitochondrial copper and that deleterious effects of ALR inhibition could be alleviated by chelating said copper with penicillamine. In contrast, we did not observe such changes in either cellular or mitochondrial copper in MitoBloCK-6 (MB-6) treated HCC cells (Supplementary Figure 5C). Due to these premises, we therefore decided not to attempt to genetically interfere with ALR action in HCC, but to use the chemical ALR inhibitor MitoBloCK-6 (MB-6), as its interference with either yeast as well as human cancer mitochondria, the latter being our research focus here, is well documented (Dabir et al., 2013; Singh et al., 2020).

Indeed, MB-6 treatment resulted in a dramatic decline in HCC proliferation, along with an increase in cellular size (Figures 1B,C). While short-term treatment over 24 h did not reveal acute cytotoxic effects, longer incubations of 48 h presented with cell death (Figures 1A,D). A decrease in cell proliferation was also recently reported in AML cells, while normal hematopoietic cells remained unaffected (Singh et al., 2020). In this same study, MB-6 was also reported to be non-toxic to mice treated with 20 doses of 80 mg/kg over 10 days. These results indicate that MB-6 might primarily affect rapidly growing cells such as tumor cells.

Most importantly, we find that pharmacological ALR inhibition in HCC cells resulted in profound mitochondrial alterations. While the mitochondrial tubular network seemed to be unaffected, electron microscopy revealed dose-dependent changes in mitochondrial ultrastructure and integrity, from apparent dilutions of cristae and the mitochondrial matrix up to outright rupture of the mitochondrial outer membranes. As a note of caution, although we cannot exclude that other ALR isoforms were equally targeted by MB-6, its specific effect on individual mitochondrial ultrastructures at its postulated site of action, i.e., the mitochondrial intermembrane space, nevertheless clearly argues for the IMS localized ALR being specifically targeted by MB-6. This conclusion is further strengthened by similar observations in yeast and human cancer mitochondria (Dabir et al., 2013; Singh et al., 2020). Moreover, the finding that the mitochondrial network was qualitatively rather unaffected may add to this conclusion, as the mitochondrial network dynamics are linked to fission/fusion, i.e., intermitochondrial events.

In-depth analysis of the mitochondrial proteome revealed broad elevations of proteins involved in mitochondrial biogenesis and homeostasis. While only minor abundance changes were observed in known DRS substrates (Supplementary Figure 1), one striking observation was the strong upregulation of proteins in the heme biosynthesis pathway, leading us to measure total and mitochondrial iron concentration as well as mitochondrial heme content (Figures 3A,B and Supplementary Figure 4A). While the iron content remained unchanged, mitochondrial heme content was significantly decreased, despite upregulation of its biosynthesis pathway. These findings indicate that, although sufficient iron and heme biosynthesis proteins are present, MB-6 nevertheless blocks heme synthesis, thereby suggesting a clear link between the mitochondrial DRS and functional iron homeostasis in HCC cells. In fact, iron depletion further potentiated the detrimental effects of MB-6 in these cells (Figure 3B and Supplementary Figure 2B), corroborating that functionally active iron (i.e., heme iron) is indeed negatively affected by MB-6.

As cancer cells typically have an increased demand for iron, possibly owed to their highly proliferative nature and increased demand for nucleotides and energy (Torti and Torti, 2013), we further followed the question whether disturbances in iron and especially heme homeostasis were a key consequence of MB-6 action in HCC cells. Thereto, we tried to rescue this inhibition by supplementing the cells with iron in the form of albumin-bound hemin b. This supplementation proved to alleviate proliferation inhibition in ALR treated cells. Furthermore, we observed almost complete restoration of mitochondrial ultrastructure, along with a profound improvement in NADH-linked mitochondrial respiration (Figure 5). To specify whether this rescue affect was specific to heme-bound iron, we subjected the cells to co-treatment with albumin-bound protoporphyrin-IX, a heme molecule lacking iron. This treatment had no positive effect on co-treated cells. We further wanted to exclude the possibility of the hemin b rescue being caused by unrelated redox-mediated effects, thus we also subjected cells to a co-treatment with albumin-bound chlorophyll b. This derivative heme molecule contains coordinated magnesium instead of iron, but had no rescue effect on MB-6 treated HCC cells. Furthermore, of these three co-treatments, only heme proved to be toxic at higher concentrations, possibly due to an eventual iron overload which is unanimously regarded as cytotoxic (Andrews, 2000).

Surprisingly, this profound rescue effect of methemalbumin supplementation did only mildly alleviate the remarkable shift in mitochondrial GSH/GSSG ratio (Supplementary Figure 4). Furthermore, we were not able to alleviate the negative effects from MB-6 treatment by providing the cells either with GSH or its precursors. This clearly suggests that it rather is the disturbed mitochondrial functional iron homeostasis and its consequent effects on respiration (e.g., via complex I) instead of oxidative stress that is the main cause for the observed cell-toxic effects of MB-6 in HCC cells. Indeed, activity measurements of diverse mitochondrial iron-containing proteins indicated impairments beyond hemoproteins. Surprisingly, complex I, a multiprotein complex which contains eight Fe-S clusters but no heme, had its activity drastically reduced by MB-6. Aconitase-2, containing both a [4Fe, 4S] cluster as well as heme, was significantly affected as well. However, complex II, containing three different Fe-S clusters as well as heme, showed no change in its activity. This latter finding could be due to a lower activity of complex II vs. I in HCC, as can be observed from high resolution respiratory measurements (Figure 5A), but may also point to a link between the DRS and heme as well as Fe-S cluster biosynthesis of selected proteins only. Indeed, the DRS has repeatedly been implicated in the maturation of Fe-S clusters, although its involvement is still a topic of debate (Crooks et al., 2018; Chang et al., 2021). Some authors have suggested a role of ALR in iron homeostasis and the maturation of cytosolic iron sulfur proteins (Lange et al., 2001), others claimed that it was neither directly nor indirectly involved. They instead suggested that defects in iron homeostasis caused by Erv1 mutations in yeast were related to a disturbed GSH status (Ozer et al., 2015). While our studies also revealed a profound shift in GSH status upon ALR inhibition, we were unable to alleviate any detrimental MB-6 effects by GSH supplementation. On the contrary, heme supplementation had a clear rescue effect, leading us to suggest the disturbed mitochondrial functional iron homeostasis as main mitochondrio-toxic effect of MB-6 in HCC. Indeed, a computational analysis recently uncovered strong co-expression between heme biosynthesis and the Fe-S cluster assembly proteins (Nilsson et al., 2009). The authors of this study described several reasons for synchronizing Fe-S cluster assembly with heme synthesis, most prominently that an Fe-S cluster is also required by mammalian ferrochelatase, which catalyzes the final step of heme synthesis. Although this cluster is absent in all prokaryotic, plant, and yeast ferrochelatases, its destruction or elimination from the mammalian enzyme results in loss of enzyme activity (Sellers et al., 1996). Intriguingly, our results further indicate that there may also be a link in the opposite direction, as heme supplementation rescued the non-heme but Fe-S cluster complex I driven respiration, which, however, remains for future studies.

In summary, our results show that MB-6, most plausibly via mitochondrial ALR inhibition, strongly reduces the growth of HCC cells and interferes with mitochondrial iron homeostasis in liver cancer cells. Furthermore, we demonstrated that these impairments can be alleviated by providing iron via heme, possibly highlighting another vital role of iron in cancer.

## Data Availability Statement

The datasets presented in this study can be found in online repositories. The names of the repository/repositories and accession number(s) can be found below: http://www.proteomexchange.org/, PXD026695.

## Author Contributions

YK designed and performed experiments, analyzed data, and wrote the manuscript. AF, AB, LJ, CE, A-CK, SH, and BM performed the experiments. TS and FB synthesized the iron chelator TS-22. PK contributed to the discussion and experimental design. HZ designed experiments, analyzed data, and wrote the manuscript. All authors have read and approved the final manuscript.

## Conflict of Interest

The authors declare that the research was conducted in the absence of any commercial or financial relationships that could be construed as a potential conflict of interest.

## Publisher’s Note

All claims expressed in this article are solely those of the authors and do not necessarily represent those of their affiliated organizations, or those of the publisher, the editors and the reviewers. Any product that may be evaluated in this article, or claim that may be made by its manufacturer, is not guaranteed or endorsed by the publisher.

## Supplementary Material

Supplementary Figure 1 Proteomic comparison of known DRS substrates. Out of 36 quantified DRS substrates, five were significantly upregulated by at least 1.5-fold, one was significantly downregulated by at least 0.5-fold and 30 proteins remained unchanged in expression levels upon MB-6 treatment.

Supplementary Figure 2 Other mitochondrial redox pairs are not affected by ALR inhibition. Deferoxamin as a further iron chelator acts similar to TS-22. **(A)** MB-6 treatment did neither change cellular NAD+/NADH ratios nor NADPH/NADP+ ratios. **(B)** Deferoxamin treatment shows clear co-toxicity together with MB-6 treatment.

Supplementary Figure 3 Methemalbumin decreases the SubG1 cell population in MB-6 treated cells. Supplementation with methemalbumin alone does not change cell cycle but distinctively reduces the SubG1 population from MB-6 treatment.

Supplementary Figure 4 Methemalbumin increases cellular and mitochondrial iron levels but does not restore GSH/GSSG levels. Copper levels are unaffected by MB-6 treatment. **(A)** Cellular and mitochondrial iron uptake by methemalbumin addition are comparable in both methemalbumin and double treated cells. **(B)** Methemalbumin supplementation slightly increases mitochondrial GSH/GSSG ratio but fails to restore impaired mitochondrial GSH/GSSG ratios in MB-6 treated cells. **(C)** Neither cellular nor mitochondrial copper content after MB-6 treatment are significantly affected. ^∗^*p* < 0.05, ^∗∗^*p* < 0.01, ^****^*p* < 0.0001.

## References

[B1] Andrews N. C. (2000). Iron metabolism: iron deficiency and iron overload. *Annu. Rev. Genomics Hum. Genet.* 1 75–98. 11701625 10.1146/annurev.genom.1.1.75

[B2] Anstee Q. M. Reeves H. L. Kotsiliti E. Govaere O. Heikenwalder M. (2019). From NASH to HCC: current concepts and future challenges. *Nat. Rev. Gastroenterol. Hepatol.* 16 411–428. 10.1038/s41575-019-0145-7 31028350

[B3] Bien M. Longen S. Wagener N. Chwalla I. Herrmann J. M. Riemer J. (2010). Mitochondrial disulfide bond formation is driven by intersubunit electron transfer in Erv1 and proofread by glutathione. *Mol. Cell* 37 516–528. 10.1016/j.molcel.2010.01.017 20188670

[B4] Chang H. C. Shapiro J. S. Jiang X. Senyei G. Sato T. Geier J. (2021). Augmenter of liver regeneration regulates cellular iron homeostasis by modulating mitochondrial transport of ATP-binding cassette B8. *eLife* 10:e65158. 10.7554/eLife.65158 33835027 PMC8055271

[B5] Crooks D. R. Maio N. Lane A. N. Jarnik M. Higashi R. M. Haller R. G. (2018). Acute loss of iron-sulfur clusters results in metabolic reprogramming and generation of lipid droplets in mammalian cells. *J. Biol. Chem.* 293 8297–8311. 10.1074/jbc.RA118.001885 29523684 PMC5971457

[B6] Dabir D. V. Hasson S. A. Setoguchi K. Johnson M. E. Wongkongkathep P. Douglas C. J. (2013). A small molecule inhibitor of redox-regulated protein translocation into mitochondria. *Dev. Cell* 25 81–92. 10.1016/j.devcel.2013.03.006 23597483 PMC3726224

[B7] Di Fonzo A. Ronchi D. Lodi T. Fassone E. Tigano M. Lamperti C. (2009). The mitochondrial disulfide relay system protein GFER is mutated in autosomal-recessive myopathy with cataract and combined respiratory-chain deficiency. *Am. J. Hum. Genet.* 84 594–604. 10.1016/j.ajhg.2009.04.004 19409522 PMC2681006

[B8] Einer C. Leitzinger C. Lichtmannegger J. Eberhagen C. Rieder T. Borchard S. (2019). A high-calorie diet aggravates mitochondrial dysfunction and triggers severe liver damage in wilson disease rats. *Cell. Mol. Gastroenterol. Hepatol.* 7 571–596. 10.1016/j.jcmgh.2018.12.005 30586623 PMC6407159

[B9] Fischer M. Riemer J. (2013). The mitochondrial disulfide relay system: roles in oxidative protein folding and beyond. *Int. J. Cell Biol.* 2013:742923. 10.1155/2013/742923 24348563 PMC3848088

[B10] Gerber J. Muhlenhoff U. Hofhaus G. Lill R. Lisowsky T. (2001). Yeast ERV2p is the first microsomal FAD-linked sulfhydryl oxidase of the Erv1p/Alrp protein family. *J. Biol. Chem.* 276 23486–23491. 10.1074/jbc.M100134200 11313344

[B11] Grosche A. Hauser A. Lepper M. F. Mayo R. Von Toerne C. Merl-Pham J. (2016). The proteome of native adult Muller Glial cells from Murine Retina. *Mol. Cell. Proteomics* 15 462–480. 10.1074/mcp.M115.052183 26324419 PMC4739667

[B12] Hofhaus G. Lee J. E. Tews I. Rosenberg B. Lisowsky T. (2003). The N-terminal cysteine pair of yeast sulfhydryl oxidase Erv1p is essential for in vivo activity and interacts with the primary redox centre. *Eur. J. Biochem.* 270 1528–1535. 10.1046/j.1432-1033.2003.03519.x 12654008

[B13] Ibrahim S. Weiss T. S. (2019). Augmenter of liver regeneration: essential for growth and beyond. *Cytokine Growth Factor Rev.* 45 65–80. 10.1016/j.cytogfr.2018.12.003 30579845

[B14] Kabiri Y. Eberhagen C. Schmitt S. Knolle P. A. Zischka H. (2021a). Isolation and electron microscopic analysis of liver cancer cell mitochondria. *Methods Mol. Biol.* 2277 277–287. 34080157 10.1007/978-1-0716-1270-5_17

[B15] Kabiri Y. Von Toerne C. Fontes A. Knolle P. A. Zischka H. (2021b). Isolation and purification of mitochondria from cell culture for proteomic analyses. *Methods Mol. Biol.* 2261 411–419. 33421004 10.1007/978-1-0716-1186-9_25

[B16] Kim E. Viatour P. (2020). Hepatocellular carcinoma: old friends and new tricks. *Exp. Mol. Med.* 52 1898–1907. 10.1038/s12276-020-00527-1 33268834 PMC8080814

[B17] Lange H. Lisowsky T. Gerber J. Muhlenhoff U. Kispal G. Lill R. (2001). An essential function of the mitochondrial sulfhydryl oxidase Erv1p/ALR in the maturation of cytosolic Fe/S proteins. *EMBO Rep.* 2 715–720. 10.1093/embo-reports/kve161 11493598 PMC1083998

[B18] Llovet J. M. Ricci S. Mazzaferro V. Hilgard P. Gane E. Blanc J. F. (2008). Sorafenib in advanced hepatocellular carcinoma. *N. Engl. J. Med.* 359 378–390. 18650514 10.1056/NEJMoa0708857

[B19] Mesecke N. Terziyska N. Kozany C. Baumann F. Neupert W. Hell K. (2005). A disulfide relay system in the intermembrane space of mitochondria that mediates protein import. *Cell* 121 1059–1069. 15989955 10.1016/j.cell.2005.04.011

[B20] Messner M. Schmitt S. Ardelt M. A. Frohlich T. Muller M. Pein H. (2020). Metabolic implication of tigecycline as an efficacious second-line Treatment for sorafenib-resistant hepatocellular carcinoma. *FASEB J.* 34 11860–11882. 10.1096/fj.202001128R 32652772

[B21] Michener J. K. Nielsen J. Smolke C. D. (2012). Identification and treatment of heme depletion attributed to overexpression of a lineage of evolved P450 monooxygenases. *Proc. Natl. Acad. Sci. U.S.A.* 109 19504–19509. 10.1073/pnas.1212287109 23129650 PMC3511110

[B22] Muchova L. Vanova K. Suk J. Micuda S. Dolezelova E. Fuksa L. (2015). Protective effect of heme oxygenase induction in ethinylestradiol-induced cholestasis. *J. Cell. Mol. Med.* 19 924–933. 10.1111/jcmm.12401 25683492 PMC4420596

[B23] Nambot S. Gavrilov D. Thevenon J. Bruel A. L. Bainbridge M. Rio M. (2017). Further delineation of a rare recessive encephalomyopathy linked to mutations in GFER thanks to data sharing of whole exome sequencing data. *Clin. Genet.* 92 188–198. 10.1111/cge.12985 28155230

[B24] Nguyen K. H. Nguyen A. H. Dabir D. V. (2017). Clinical implications of augmenter of liver regeneration in cancer: a systematic review. *Anticancer Res.* 37 3379–3383. 10.21873/anticanres.11704 28668825

[B25] Nilsson R. Schultz I. J. Pierce E. L. Soltis K. A. Naranuntarat A. Ward D. M. (2009). Discovery of genes essential for heme biosynthesis through large-scale gene expression analysis. *Cell Metab.* 10 119–130. 10.1016/j.cmet.2009.06.012 19656490 PMC2745341

[B26] Ozer H. K. Dlouhy A. C. Thornton J. D. Hu J. Liu Y. Barycki J. J. (2015). Cytosolic Fe-S cluster protein maturation and iron regulation are independent of the mitochondrial Erv1/Mia40 import system. *J. Biol. Chem.* 290 27829–27840. 10.1074/jbc.M115.682179 26396185 PMC4646028

[B27] Perez-Riverol Y. Csordas A. Bai J. Bernal-Llinares M. Hewapathirana S. Kundu D. J. (2019). The PRIDE database and related tools and resources in 2019: improving support for quantification data. *Nucleic Acids Res.* 47 D442–D450. 10.1093/nar/gky1106 30395289 PMC6323896

[B28] Pozarowski P. Darzynkiewicz Z. (2004). Analysis of cell cycle by flow cytometry. *Methods Mol. Biol.* 281 301–311. 15220539 10.1385/1-59259-811-0:301

[B29] Rahman I. Kode A. Biswas S. K. (2006). Assay for quantitative determination of glutathione and glutathione disulfide levels using enzymatic recycling method. *Nat. Protoc.* 1 3159–3165. 17406579 10.1038/nprot.2006.378

[B30] Riddle R. D. Yamamoto M. Engel J. D. (1989). Expression of delta-aminolevulinate synthase in avian cells: separate genes encode erythroid-specific and nonspecific isozymes. *Proc. Natl. Acad. Sci. U.S.A.* 86 792–796. 10.1073/pnas.86.3.792 2915978 PMC286563

[B31] Rissler M. Wiedemann N. Pfannschmidt S. Gabriel K. Guiard B. Pfanner N. (2005). The essential mitochondrial protein Erv1 cooperates with Mia40 in biogenesis of intermembrane space proteins. *J. Mol. Biol.* 353 485–492. 10.1016/j.jmb.2005.08.051 16181637

[B32] Schmitt S. Saathoff F. Meissner L. Schropp E. M. Lichtmannegger J. Schulz S. (2013). A semi-automated method for isolating functionally intact mitochondria from cultured cells and tissue biopsies. *Anal. Biochem.* 443 66–74. 23969012 10.1016/j.ab.2013.08.007

[B33] Schulz S. Schmitt S. Wimmer R. Aichler M. Eisenhofer S. Lichtmannegger J. (2013). Progressive stages of mitochondrial destruction caused by cell toxic bile salts. *Biochim. Biophys. Acta* 1828 2121–2133. 10.1016/j.bbamem.2013.05.007 23685124

[B34] Schulz T. J. Thierbach R. Voigt A. Drewes G. Mietzner B. Steinberg P. (2006). Induction of oxidative metabolism by mitochondrial frataxin inhibits cancer growth: Otto Warburg revisited. *J. Biol. Chem.* 281 977–981. 10.1074/jbc.M511064200 16263703

[B35] Sellers V. M. Johnson M. K. Dailey H. A. (1996). Function of the [2FE-2S] cluster in mammalian ferrochelatase: a possible role as a nitric oxide sensor. *Biochemistry* 35 2699–2704. 10.1021/bi952631p 8611576

[B36] Singh R. P. Jeyaraju D. V. Voisin V. Hurren R. Xu C. Hawley J. R. (2020). Disrupting mitochondrial copper distribution inhibits Leukemic stem cell self-renewal. *Cell Stem Cell* 26 926–937.e10. 10.1016/j.stem.2020.04.010 32416059

[B37] Spinazzi M. Casarin A. Pertegato V. Salviati L. Angelini C. (2012). Assessment of mitochondrial respiratory chain enzymatic activities on tissues and cultured cells. *Nat. Protoc.* 7 1235–1246. 22653162 10.1038/nprot.2012.058

[B38] Thomas L. W. Ashcroft M. (2019). Exploring the molecular interface between hypoxia-inducible factor signalling and mitochondria. *Cell. Mol. Life Sci.* 76 1759–1777. 10.1007/s00018-019-03039-y 30767037 PMC6453877

[B39] Torti S. V. Torti F. M. (2013). Iron and cancer: more ore to be mined. *Nat. Rev. Cancer* 13 342–355. 23594855 10.1038/nrc3495 PMC4036554

[B40] van Malenstein H. Dekervel J. Verslype C. Van Cutsem E. Windmolders P. Nevens F. (2013). Long-term exposure to sorafenib of liver cancer cells induces resistance with epithelial-to-mesenchymal transition, increased invasion and risk of rebound growth. *Cancer Lett.* 329 74–83. 10.1016/j.canlet.2012.10.021 23111106

[B41] Ward P. S. Thompson C. B. (2012). Metabolic reprogramming: a cancer hallmark even warburg did not anticipate. *Cancer Cell* 21 297–308. 22439925 10.1016/j.ccr.2012.02.014 PMC3311998

[B42] Winterbourn C. C. (1995). Toxicity of iron and hydrogen peroxide: the Fenton reaction. *Toxicol. Lett.* 8 969–974. 10.1016/0378-4274(95)03532-x 8597169

[B43] Wisniewski J. R. Zougman A. Nagaraj N. Mann M. (2009). Universal sample preparation method for proteome analysis. *Nat. Methods* 6 359–362. 19377485 10.1038/nmeth.1322

[B44] Yu H. Y. Zhu M. H. Xiang D. R. Li J. Sheng J. F. (2014). High expression of 23 kDa protein of augmenter of liver regeneration (ALR) in human hepatocellular carcinoma. *Onco Targets Ther.* 7 887–893. 10.2147/OTT.S61531 24940072 PMC4051792

[B45] Zischka H. Larochette N. Hoffmann F. Hamoller D. Jagemann N. Lichtmannegger J. (2008). Electrophoretic analysis of the mitochondrial outer membrane rupture induced by permeability transition. *Anal. Chem.* 80 5051–5058. 10.1021/ac800173r 18510346

[B46] Zischka H. Lichtmannegger J. Schmitt S. Jagemann N. Schulz S. Wartini D. (2011). Liver mitochondrial membrane crosslinking and destruction in a rat model of Wilson disease. *J. Clin. Invest.* 121 1508–1518. 10.1172/JCI45401 21364284 PMC3068979

