# Supplementary figures and images for: Mitochondrial Impairment by MitoBloCK-6 Inhibits Liver Cancer Cell Proliferation
Source: Front Cell Dev Biol. 2021 Sep 20;9:725474. doi: 10.3389/fcell.2021.725474 (PMC8488156; doi:10.3389/fcell.2021.725474)

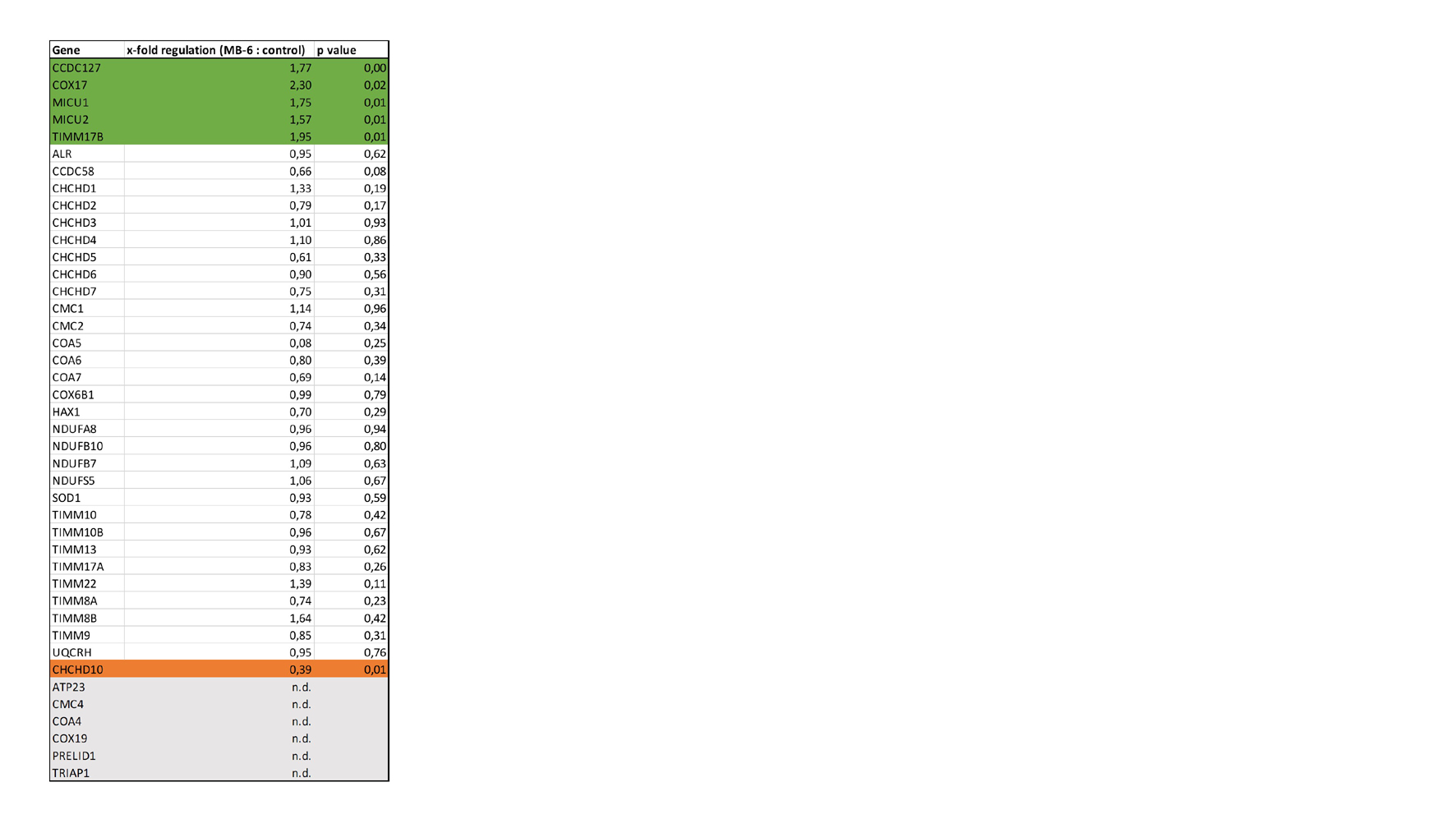

Supplement: Supplementary Figure 1 — Proteomic comparison of known DRS substrates. Out of 36 quantified DRS substrates, five were significantly upregulated by at least 1.5-fold, one was significantly downregulated by at least 0.5-fold and 30 proteins remained unchanged in expression levels upon MB-6 treatment. [file Image_1.JPEG]

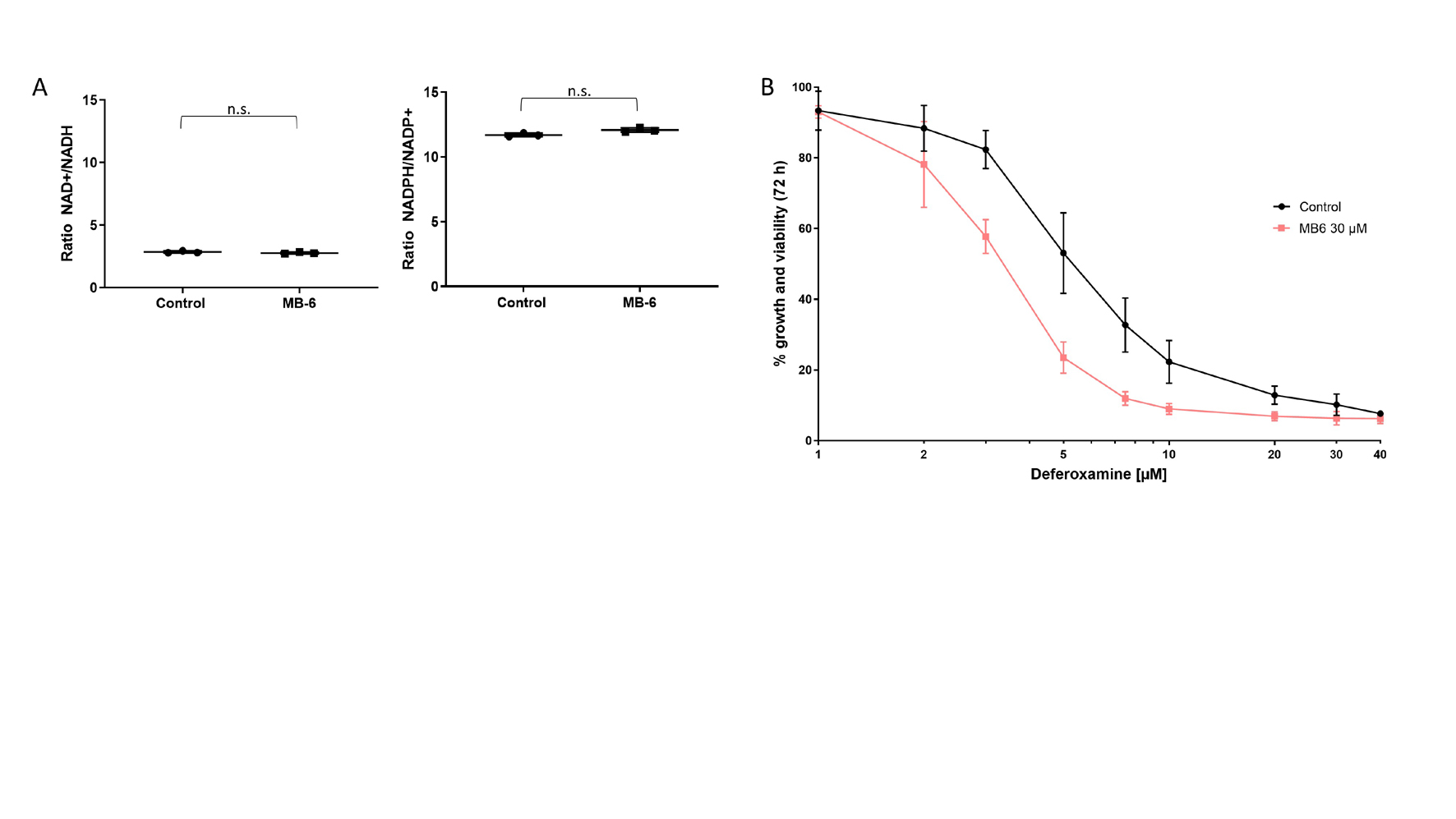

Supplement: Supplementary Figure 2 — Other mitochondrial redox pairs are not affected by ALR inhibition. Deferoxamin as a further iron chelator acts similar to TS-22. (A) MB-6 treatment did neither change cellular NAD+/NADH ratios nor NADPH/NADP+ ratios. (B) Deferoxamin treatment shows clear co-toxicity together with MB-6 treatment. [file Image_2.JPEG]

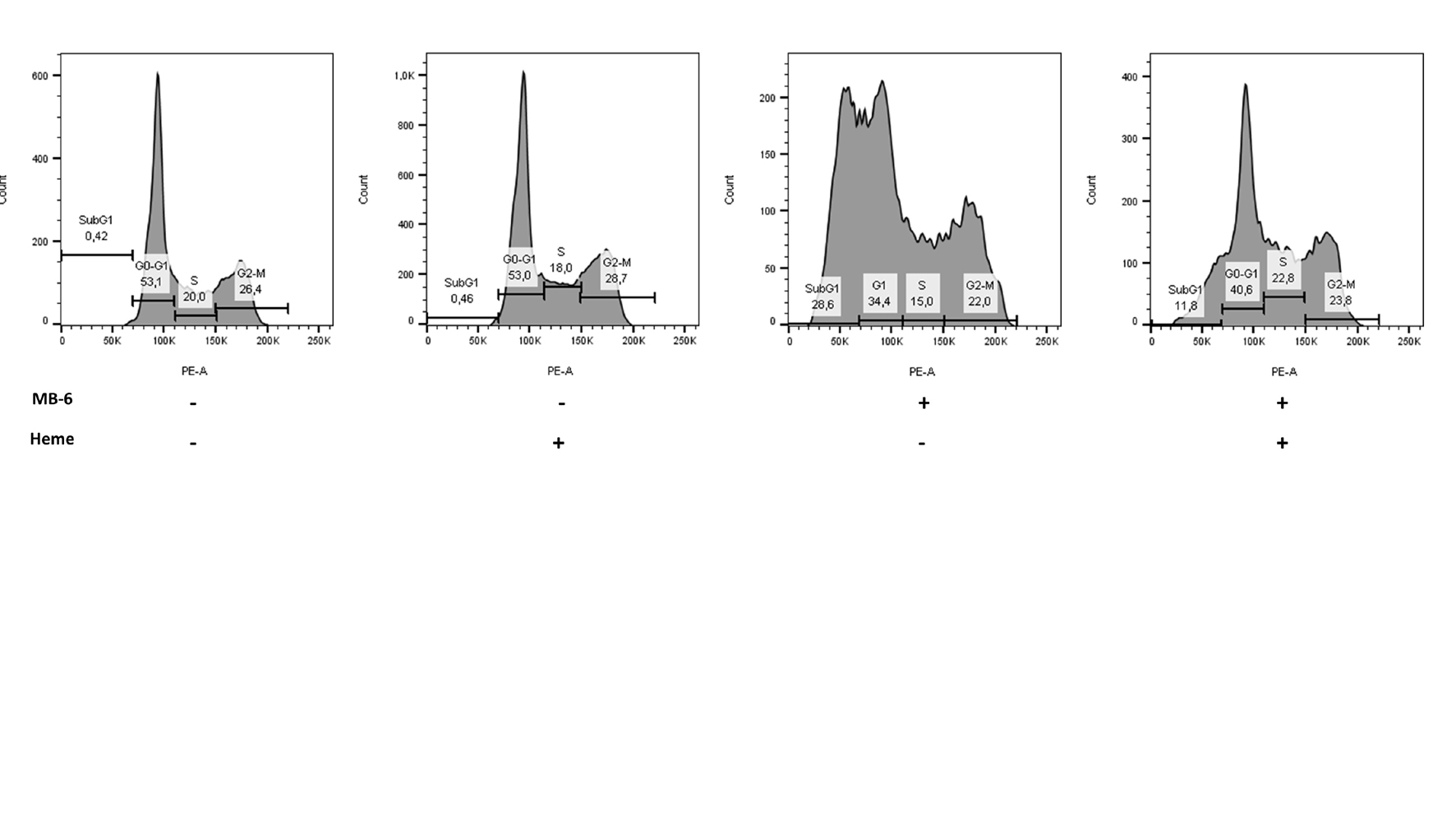

Supplement: Supplementary Figure 3 — Methemalbumin decreases the SubG1 cell population in MB-6 treated cells. Supplementation with methemalbumin alone does not change cell cycle but distinctively reduces the SubG1 population from MB-6 treatment. [file Image_3.JPEG]

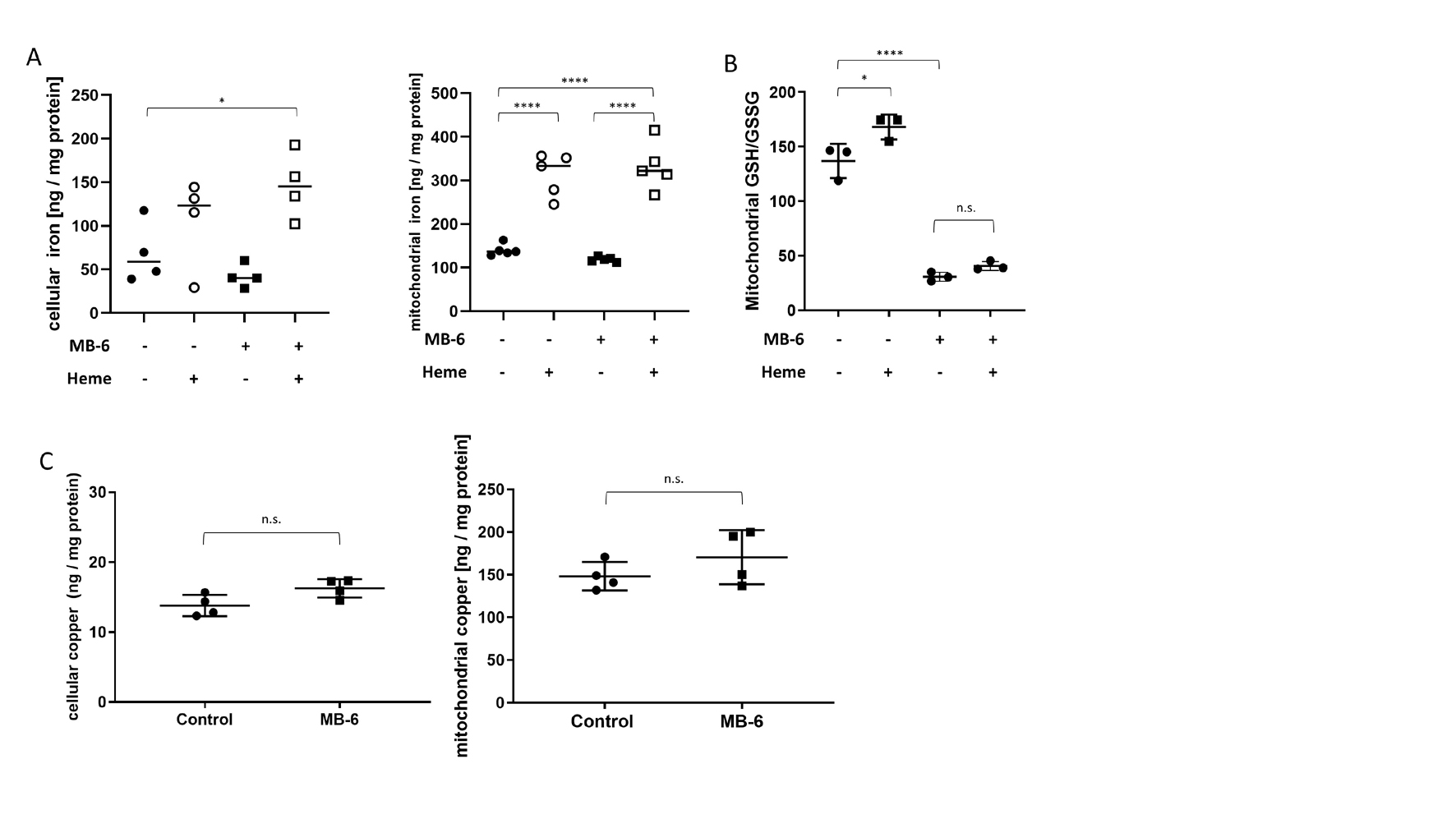

Supplement: Supplementary Figure 4 — Methemalbumin increases cellular and mitochondrial iron levels but does not restore GSH/GSSG levels. Copper levels are unaffected by MB-6 treatment. (A) Cellular and mitochondrial iron uptake by methemalbumin addition are comparable in both methemalbumin and double treated cells. (B) Methemalbumin supplementation slightly increases mitochondrial GSH/GSSG ratio but fails to restore impaired mitochondrial GSH/GSSG ratios in MB-6 treated cells. (C) Neither cellular nor mitochondrial copper content after MB-6 treatment are significantly affected. ∗p < 0.05, ∗∗p < 0.01, ****p < 0.0001. [file Image_4.JPEG]
